# Supplementary material for: Individual and Community-Level Risk Factors for Giardiasis in Children under Five Years of Age in Pakistan: A Prospective Multi-Regional Study
Source: Children (Basel). 2023 Jun 20;10(6):1087. doi: 10.3390/children10061087 (PMC10297578; doi:10.3390/children10061087)
Supplement: Supplementary file 1 [file children-10-01087-s001.zip › children-2376511-supplementary.pdf]

Supplementary File S1

Questionnaire

Title: A study of individual and community-level risk factors for giardiasis in children under five in Khyber Pakhtunkhwa, Pakistan

Institute: Abdul Wali Khan University, Mardan, KP, Pakistan

Study Period: March to December 2022

Outcome: To determine the prevalence of *G. lamblia* infection in a healthy pediatric population across several agro-ecological zones of Khyber Pakhtunkhwa, Pakistan, and to identify any potential individual and community-level risk factors.

PersonalInformation

Respondent's ID________________ **Sample No._________________**

Respondent's Name_____________ **Father Name: ________________**

Gender: __________________

Age (months):

- 0-6
- 7-12
- 13-24
- 25-36
- 37-48
- 49-60

Family size

- <6
- >6

Mother's educational level

- Can't read and write
- Primary
- Secondary
- Higher education

Father's educational level

- Can't read and write
- Primary
- Secondary
- Higher education

Attend day care institution/Kindergarten

- Yes
- No

Handwashing behavior

- Washes at critical times
- Does not wash at critical times

Companion animal

- Yes
- No

Stray dogs/ cats enter home

- Yes
- No

Agro-ecological zones

- High dry mountains
- Sub mountain valleys
- Sub humid mountains
- Wet mountains
- Valley plains
- Piedmont plain
- Semi-arid piedmont
- Western mountains
- Desert plains

Drinking water sources

- Piped
- Un-piped

Domestic water storage vessel

- Adequate
- Inadequate

Drinking water boiled or filtered

- Yes
- No

Presence of rubbish heap near house

- Not present
- Present

Condition of street or pathway

- Paved
- Unpaved

Latrine facilities

- Improved
- Unimproved

Patient'sconsent

I Mr/Mrs/Miss________________ Guardian of _________________give my consent for personal and clinical information related to the child that this data will be used for research purpose and will be published without our personal identity.

Signature of Child Guardian: __________________

Date:________________
